# LncRNAs in Cancer: From garbage to Junk

**DOI:** 10.3390/cancers12113220

**Published:** 2020-10-31

**Authors:** Marianna Aprile, Vicky Katopodi, Eleonora Leucci, Valerio Costa

**Affiliations:** 1 Institute of Genetics and Biophysics “Adriano Buzzati-Traverso”, CNR, 80131 Naples, Italy; marianna.aprile@igb.cnr.it; 2 Laboratory for RNA Cancer Biology, Department of Oncology, KULeuven, LKI, Herestraat 49, 3000 Leuven, Belgium; vasiliki.katopodi@kuleuven.be (V.K.); eleonora.leucci@kuleuven.be (E.L.)

**Keywords:** translational reprogramming, oncogenic lncRNAs, tumor suppressor lncRNAs, therapeutic targeting, epigenetics

## Abstract

**Simple Summary:**

Long non-coding RNAs (lncRNAs) are generally defined as transcripts longer than 200 nucleotides and not coding for proteins. This review provides an overview of the main functions of lncRNAs in the cells and their role in tumorigenesis. Additionally, key examples of oncogenic and/or tumor suppressive lncRNAs with a well-established role in tumor development and progression are discussed. Finally, the currently available technological approaches to target lncRNAs (e.g., by modifying their expression, stability, processing and/or binding capacity), with a specific focus on the pros and cons of their use as therapeutic targets are considered.

**Abstract:**

Sequencing-based transcriptomics has significantly redefined the concept of genome complexity, leading to the identification of thousands of lncRNA genes identification of thousands of lncRNA genes whose products possess transcriptional and/or post-transcriptional regulatory functions that help to shape cell functionality and fate. Indeed, it is well-established now that lncRNAs play a key role in the regulation of gene expression through epigenetic and posttranscriptional mechanims. The rapid increase of studies reporting lncRNAs alteration in cancers has also highlighted their relevance for tumorigenesis. Herein we describe the most prominent examples of well-established lncRNAs having oncogenic and/or tumor suppressive activity. We also discuss how technical advances have provided new therapeutic strategies based on their targeting, and also report the challenges towards their use in the clinical settings.

## 1. Introduction

In the human genome, the protein-coding genes represent less than 2%, whereas a large fraction is constituted by regions—often transcribed on both DNA strands—whose products lack any significant translational potential [1]. In the last two decades, some gaps in the knowledge of the genomic complexity has been filled in by the identification of several families of long RNAs. In particular, long non-coding RNAs (lncRNAs)—some of which already had attributed gene-specific regulatory functions as early as the 1990s (e.g., *H19* and *XIST*)—are arbitrarily defined as RNAs longer than 200 nucleotides lacking coding potential. They constitute a highly heterogeneous family of ncRNAs transcribed by RNA polymerase II and arise from intergenic (gene deserts), as well as intronic or gene-dense regions [2]. Generally, lncRNAs are 5’ capped and 3’ polyadenylated and often undergo splicing similarly to mRNAs [3,4]. Sequencing-based transcriptomics approaches—and especially RNA sequencing—have completely redefined the picture of human genome transcription, enabling the identification of a very large number of lncRNA genes [5,6,7]. Indeed, more than 48,600 transcripts from about 18,000 genes in the human transcriptome are annotated as bona fide lncRNAs (evidence-based annotation of the human genome release GRCh38, GENCODE version 35).

Despite the rapid advancement in their identification and expression analysis, the functional characterization of lncRNA is still lagging behind, possibly because, unlike other ncRNAs, they can employ a wide array of functions. They can act as decoys, guides, and scaffolds, exerting transcriptional and/or post-transcriptional regulatory activities both in the nucleus and in the cytoplasm by direct or indirect interactions with chromatin, proteins, and other RNAs [8]. Therefore, lncRNAs participate virtually in all relevant cellular processes (e.g., maintenance of stemness, proliferation, angiogenesis, apoptosis etc.), both in physiological and pathological conditions and, not surprisingly, alterations in lncRNA expression have been frequently associated with cancer onset and progression [9].

Among the large-scale sequencing projects aiming to characterize cancer genomes, the Cancer Genome Atlas (TCGA) Consortium has provided a wide molecular characterization of about 11,000 primary cancers, uncovering a substantial fraction of new somatic alterations (i.e., point mutations and/or small indels, gene rearrangements, and copy-number alterations). Khurana and colleagues reported that ~99% of somatic SNVs in tumors occur in noncoding regions, which include transcription factor binding sites (TFBS), ncRNAs, and pseudogenes [10]. A recent study based on TCGA and lncRNA expression data from TANRIC [11] reveals that mutational frequencies in lncRNAs whose expression is affected by somatic alterations (MutLncs) are low and that, to a certain extent, their alteration tends to be cancer type-specific [12]. Moreover, a large proportion of mutations are located within lncRNA TFBS, especially of ESR1, TRPS1, ERG, and RUNX1.

A growing number of studies reveals a previously unrecognized role for lncRNAs as conditional and constitutive oncogenes and tumor suppressors by means of their ability to regulate each and every cancer hallmark (e.g., uncontrolled proliferation, metastasis, immune escape, etc.) [13,14,15,16,17]. These findings, and especially the cancer-specific expression of most of them, established the rationale for assessing lncRNAs as possible biomarkers and/or therapeutic targets, as their silencing would not cause side-effects on other tissues.

This review provides an overview of the main regulatory functions of lncRNAs in tumorigenesis, their alteration in different cancer types and the main strategies to target them. Furthermore, we discuss future directions in cancer research based on the emerging lncRNA properties.

## 2. Epigenetic Regulation and Translational Reprogramming

Compartmentalization in time and space [18,19,20] offered the first indication that lncRNAs may not be mere transcriptional noise, but they could be implicated in virtually all fundamental cellular processes. Indeed, disproving the notion that sequence conservation is essential to postulate a function, the number of lncRNAs with attributed regulatory roles is exponentially increasing [21,22]

With a few exceptions, lncRNAs are generally poorly expressed [18,20] and dispensable for organismal viability in physiological conditions [23,24,25]; however, they have been implicated in all known stress response pathways [26,27]. As such, they play crucial roles in the epigenetic and post-transcriptional regulation of gene expression. 

Nuclear lncRNAs have broadened our knowledge of transcriptional regulation from a transcription factor-centric to a more complex view that integrates protein- and RNA-based regulation. The latter controls histone and DNA modifications and the transcriptional machinery by recruiting specific factors to the DNA or sponging them away from it. In this way they contribute both to the regulation of a large number of genes or of specific loci [28]. 

Cytoplasmic lncRNAs, which are proportionally more abundant than nuclear ones [29], have emerged in the past decade as modulators of RNA stability, localization, and translation. Apart from interaction with the translational machinery, which was revealed by ribosome profiling and translating ribosome affinity purification (TRAP) approaches [30,31], cytoplasmic lncRNAs can also sponge proteins or other RNA molecules, serving as competing-endogenous RNAs (ceRNAs). The discovery of lncRNAs has therefore added a layer of complexity to cytoplasmic post-transcriptional and translational control, dominated before by proteins and miRNAs [32]. 

### 2.1. LncRNAs in Epigenetic and Transcriptional Regulation (DNA Methylation, Nucleosome Positioning, and Histone Modifications)

**Figure 1:**
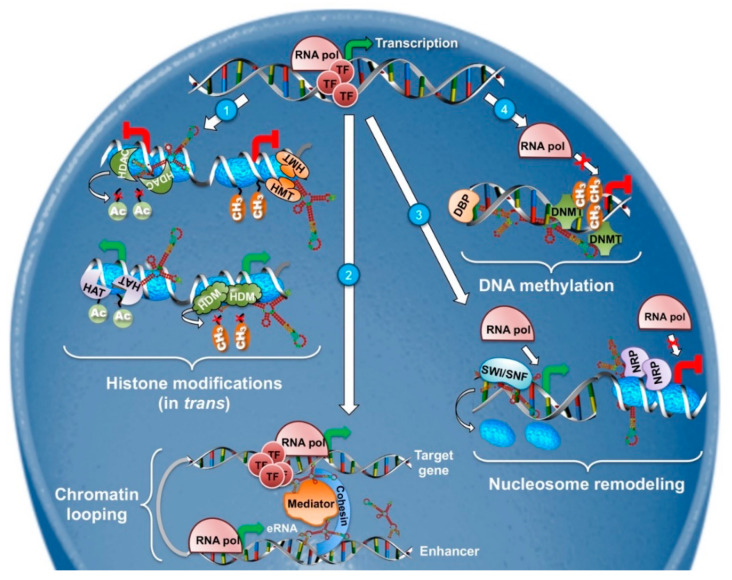
LncRNAs in epigenetic regulation of gene expression. Schematic models of (1) *trans*-acting lncRNAs recruiting histone deacetylases (HDACs, **upper left**), histone methyltransferases (HMTs, **upper right**) associated to gene repression, or histone acetylases (HATs, **lower left**) and histone demethylases (HDMs, **lower right**) associated to transcription-permissive chromatin; (2) enancher RNA (eRNA) recruiting the Mediator complex—with cohesin-based stabilizing contacts—responsible for DNA looping and distal transcriptional activation of target genes; (3) lncRNAs interacting with nucleosome destabilizing proteins, such as the SWI/SNF complex (**left**), causing nucleosome exclusion and gene activation, or with remodeling proteins (NRPs, **right**) associated with chromatin condensation and transcriptional repression; and (4) lncRNA acting as a scaffold for DNA methyltransferases (DNMTs) and DNA-binding proteins (DBP), and driving gene promoters’ methylation and their transcriptional repression.

Historically, lncRNA implication in nuclear activities such as epigenetics and transcriptional regulation was the first to be recognized and it is probably the best characterized. LncRNAs can regulate the expression of protein-coding genes in close proximity (*cis*-acting) or even influence distant transcriptional activators and repressors (*trans*-acting) (Figure 1) [33]. Their mechanisms of action extend from chromatin remodeling, to binding and regulating transcription factors and epigenetic regulators and in some instances, the sole act of their transcription is sufficient to exert a regulatory function [34,35].

#### 2.1.1. DNA Methylation

DNA methylation is a fundamental epigenetic process regulating gene expression and is orchestrated by several DNA methyltransferases. Changes in DNA methylation patterns are crucial for normal development [36,37] but also for cancer. Aberrant DNA hyper- or hypomethylation regulates the expression of key oncogenes and tumor suppressors and affects genome stability, thus directly participating in cancer development and progression [38]. Since the discovery of lncRNAs, increasing evidence has been linking them to the regulation of DNA methylation. For instance, *ecCEBP* lncRNA was shown to directly interact with DNA-methyltransferases (DNMTs) preventing DNA methylation at the *CENPA* locus [39].

Later on, 148 other lncRNAs were identified as interactors of DNMT1 in colon cancer cells by RIP-seq [40]. One of them, *DACOR1* (DNMT1-associated colon cancer repressed lncRNA 1) is highly expressed in normal colon but is suppressed in various colon tumors and patient-derived cancer cell lines. Expression of *DACOR1* is sufficient to restore the DNA methylation status at thousands of CpG sites hypomethylated in colorectal cancer [41]. Furthermore, *DACOR1* induction reduces colon cancer clonogenic ability, thus supporting a tumor-suppressor function for this lncRNA [40]. 

The emerging widespread indications that lncRNAs can influence DNA-methylation have recently been gathered in the Lnc2Meth database. This web tool aims to close the gap on the regulatory relationships between DNA-methylation and lncRNAs with experimentally verified information [42]. 

#### 2.1.2. Histone Modifications

Apart from DNA methylation, chromatin structure -and thus transcription- can be influenced by post-translational modifications of histone proteins. Histones are subject to numerous modifications catalyzed by specific histone-modifying enzymes, including acetylation and methylation of lysine, methylation of arginine, phosphorylation of serine and threonine, ubiquitylation of lysine residues, glycosylation, sumoylation, adenosine diphosphate ribosylation, and carbonylation [43]. Demethylation and histone acetylation, for example, mediated by histone demethylases (HDMs) and histone acetyltransferases (HATs), respectively, favors chromatin decondensation, whereas trimethylation of lysine 27 on histone 3 by the polycomb system, as well as deacetylation by histone deacetylases (HDACs), are implicated in repressive chromatin (schematized in Figure 1). Specific patterns of local and global histone modifications are required for the maintenance of the cells identity and alterations have been linked to cancer formation [44,45,46]. Apart from the modifications themselves, deregulation of the enzymes responsible for them have also been documented in many cancers [47,48], highlighting the importance of this epigenetic mechanism.

Although lncRNAs have been only recently associated with histone modifying enzymes [49,50,51] a paradigm of this category has long been known. X-inactive–specific transcript (*XIST*) RNA was one of the first long non-coding RNAs to be discovered in the early 1990s [52,53,54]. As its name dictates, *XIST* is a master regulator of the X chromosome inactivation (XCI) in the female mammal, a process meant to balance X-chromosome gene expression between the two sexes [55,56]. The initiation of XCI depends on *XIST*, which tethers polycomb-repressive complexes to the X chromosome *in cis*, in order to trimethylate histone H3 on lysine 27 (H3K27me3) resulting in transcriptional silencing. The tremendous effects of *XIST* on chromatin formation result in the conversion of an entire chromosome into a unique heterochromatic entity known as the Barr body [57]. After the completion of XCI, *XIST* is no longer needed but continues to be expressed, albeit at low levels, throughout female life [58]. X chromosomes’ aneuploidies have been associated with human cancers for a long time [59,60] and Lee’s lab proved the role of *XIST* in this process. Deletion of *Xist* was shown to be sufficient to induce a female-specific, aggressive and ultimately lethal blood cancer, possibly due to upregulation of X-linked genes [58].

Another example of lncRNAs exerting their regulatory functions through local chromatin remodeling is *ANRIL* (antisense non-coding RNA in the INK4 locus). *ANRIL* is a 3834 bp long transcript in the *INK4A-ARF-INK4B* gene cluster. Visel and colleagues provided the first genetic evidence of a negative regulation of *p16^INK4A^* and *p15^INK4B^* by non-coding RNAs residing in this genomic region [61]. Later on, it was demonstrated that *ANRIL* recruits the Polycomb repressive complex 2 (PRC2), well-known for its histone methyltransferase (HMT) activity on histone 3 lysine 27, to the *p15^INK4B^* locus [62]. *ANRIL* acts as a molecular scaffold which physically interacts with SUZ2, a PRC2 component, forcing the complex to occupy and thus regulate the *p15^INK4B^* locus. As one of the most important tumor suppressor loci, the *INK4A-ARF-INK4B* gene cluster is frequently deleted or silenced in many human cancers, making *ANRIL* one of the most altered lncRNAs [63,64,65,66].

Epigenetic silencing mediated by the interaction of lncRNAs with PRC2 has been proposed as a general mechanism for numerous nuclear lncRNAs [49,67,68]. However, several studies showing PRC2 promiscuous interaction with more RNA species [69,70,71], challenge the specificity and functional importance of lncRNA-PRC2 interactions. Indeed, Portoso and colleagues demonstrated that *HOTAIR* (Hox transcript antisense intergenic RNA) lncRNA, previously shown to interact with PRC2 in order to exert its transcriptional repressive functions on the *HOXD* locus [72], does not depend on PRC2 for the deposition of H3K27 trimethylation marks. On the contrary, the expression and correct localization of *HOTAIR* are necessary for silencing [73]. These results support the notion that even if a lncRNA-PRC2 complex forms, it might not, itself, be directly implicated in gene regulation and call for critical evaluation of the functionality of such complexes.

Finally, although less studied, a few lncRNAs have been associated with regulation of other histone marks. An example is lncPRESS1 which is a p53 target that disrupts SIRT6-mediated H3K56 deacethylation. Although the role of lncPRESS1 in cancer has not been investigated yet, it is very likely considering that it is associated with the guardian of the genome [74].

#### 2.1.3. Chromatin Looping

Transcriptional gene regulation can also be achieved at a higher level of DNA compaction through the control of chromatin looping and nucleosome positioning. The effects of chromatin looping on transcription regulation can be probably best described by active enhancers regulating distant genes. Enhancers are tissue-specific DNA elements of low sequence and high functional conservation [75]. They bind transcription factors and mediator complex to recruit Polymerase II and enforce chromatin loops to increase the rate of transcription at distal promoters [76]. Active enhancers are pervasively transcribed giving rise to cell type- and state-specific transcripts called eRNAs [77,78]. eRNAs are non-coding RNAs that can be categorized into short, bidirectional, non-polyadenylated, mono-exonic transcripts or longer, unidirectional, polyadenylated, and spliced, both of which are of low abundance and enriched in the nucleus [78]. Whether eRNAs themselves are needed for a functional enhancer is still under debate; nevertheless, many studies show that eRNAs can modulate chromatin by stabilizing enhancer-promoter looping (Figure 1) [77,79,80]. Under normal conditions active enhancers control the maintenance of different cell types and it is of no surprise that they are deregulated in many human cancers [81]. Along with them, eRNAs are differentially expressed in cancer tissues and were shown to act as oncogenes, promoting the transcriptional activation of oncogenic networks and even inducing chromosome rearrangements and genomic instability [82,83,84]. 

The eRNA-acting lncRNA Leukemia-induced Non-coding Activator RNA-1 (*LUNAR1*) was initially identified by RNA-Seq in T-cell leukemia [85]. *LUNAR1* is a T-ALL specific, Notch-dependent, nuclear-enriched lncRNA, whose coding neighbor is the insulin-like growth factor receptor 1 (*IGF1R*) implicated in T-ALL initiation and Notch signaling [86]. Depletion of *LUNAR1* leads to downregulation of *IGF1R* and significant reduction in Mediator Complex and RNA Pol II occupancy at both the *IGF1R* enhancer and promoter. Hi-C experiments and 3C-qPCR showed that *LUNAR1* is physically associated with the Notch-occupied IGF1R enhancer and with *LUNAR1* promoter, proving that this lncRNA exploits chromatin looping to reach to its target and then recruits Mediator to activate the target-promoter [85].

#### 2.1.4. Nucleosome Positioning

Nucleosome positioning is a major factor in controlling gene expression since tight interaction of nucleosomes with histone cores can strongly affect DNA accessibility [87]. LncRNAs can regulate nucleosome positioning in various ways ranging from the recruitment of ATP-dependent remodelers to transcription mediated nucleosome stabilization (Figure 1) [88]. The human lncRNA *SChLAP1* controls nucleosome positioning via negative regulation of ATP-dependent chromatin remodelers. It physically interacts with the SNF5-subunit of the SWI/SNF chromatin-remodeling complex, responsible for nucleosome positioning, and prevents its binding to the chromatin, thus regulating gene expression. SChLAP1 has high prognostic value for prostate cancer and it is the first documented lncRNA acting as an antagonist of a major epigenetic complex, making it a great example for lncRNAs regulating nucleosome positioning [89]. 

Another lncRNA that exemplifies the complexity of lncRNAs functions in nucleosome positioning is promoter and pre-rRNA antisense *PAPAS* [90]. *PAPAS* links histone modifications together with nucleosome remodeling, ribosome biogenesis and by extension protein synthesis. *PAPAS* is an antisense RNA to pre-rRNA genes which inhibits pre-rRNA synthesis under growth-factor deprivation [91]. This lncRNA is upregulated in starving cells and acts as a scaffold for the histone methyltransferase Suv4-20h2, guiding it to rRNA genes and leading to trimethylation of histone H4 at lysine 20 (H4K20me3) and chromatin compaction. In this way, rRNA promoters become inaccessible to RNA polymerase I, resulting in ribosome biogenesis attenuation [91]. Interestingly enough, upon hypotonic stress *PAPAS* still suppresses transcription of rRNA genes but through a different mechanism. In these conditions Suv4-20h2 gets ubiquitinylated and degraded, therefore *PAPAS* interacts instead with CHD4, a subunit of the nucleosome remodeling and deacetylation complex (NuRD). This interaction causes a shift of the promoter-bound nucleosome into a position that blocks transcription initiation [92,93].

Whether this lncRNA is deregulated in cancer is not yet known to our knowledge, however, *PAPAS’* functions prove not only that transcriptional control by lncRNAs can be facilitated through many mechanisms but also that a single lncRNA can have different modes of action depending on the cell state.

### 2.2. LncRNA Acting as ceRNAs

LncRNAs can serve as master regulators at the post-transcriptional level, acting as competing endogenous RNAs (ceRNAs). ceRNAs regulate other (m)RNA targets by competing for the binding of shared miRNAs [94,95]. LncRNA-bound miRNAs can no longer be loaded to the AGO2/RISC and are, thus, unable to repress the translation of their mRNA-targets. Through this miRNA-mediated mode of action, ceRNAs emerge as pluripotent regulators capable of influencing an entire post-transcriptional regulatory network mediated by multiple miRNAs. Their importance is further proved by studies conducted in solid tumors and hematopoietic malignancies where ceRNAs alter the expression of tumor suppressors and oncogenes, in favor of cancer progression [96]. 

The first example of a non-coding transcript acting as a miRNA-sponge was provided by a processed pseudogene, highly homologous to the *PTEN* gene, called *PTENP1* (phosphatase and tensin homolog pseudogene 1) that harbored several binding sites for *PTEN*-targeting miRNAs. In prostate cancer, the competition between *PTENP1* and *PTEN* for binding with their shared miRNAs, positively regulates *PTEN* protein levels, placing *PTENP1* in the category of tumor-suppressor lncRNAs [97]. Following their discovery, *PTENP1s* tumor-suppressor functions were confirmed in many other cancer types [98,99,100] proving beyond doubt that lncRNAs can exert ceRNA-related functions of great impact. 

Another paradigm of this function is *HULC,* a very abundant lncRNA in hepatocellular carcinoma, and many other cancer types, that resembles the mammalian LTR transposon 1A [101]. Acting as an oncogene, it promotes tumor angiogenesis, cell proliferation, and abnormal lipid metabolism, by multiple mechanisms, such as the regulation of sphingosine kinase 1 expression and PI3K/AKT signaling [102,103,104]. The expression levels of *HULC* in cancer patients have been correlated with their clinical outcome [105]. Furthermore, acting as a ceRNA, *HULC* ensures its own high expression levels by sponging miR-372 from binding and suppressing translation of *PRKACB. PRKACB*, in turn induces the phosphorylation of cAMP response element binding protein (CREB) leading to upregulation of *HULC* transcription [106]. 

### 2.3. LncRNAs in Translational Reprogramming

Gene expression regulation is not limited to transcriptional and epigenetic regulatory networks. Protein synthesis is the most energy-demanding process inside the cell and, therefore, it is very tightly regulated [26]. Accordingly, this process is controlled by all the major tumor suppressors and oncogenes at all stages starting from rRNA biosynthesis in the nucleoli and ending with ribosome recycling [107]. Regulation at the translational level offers the advantage to rapidly respond to environmental changes [108]. 

LncRNAs have been associated with all the steps of protein synthesis, the simplest way being repressing or promoting the translation of specific mRNA-targets. This mode of action can be exemplified by the human lncRNA *lincRNA-p21*, a cytoplasmic-enriched molecule known to co-localize with ribosomes. In human cervical carcinoma (HeLa) cells *lincRNA-p21* was shown to be negatively regulated by the RNA binding protein Hu antigen R (HuR) and, only when HuRs levels decrease, the lncRNA is able to stabilize and interact with its mRNA-targets: mainly *CTNNB1* and *JUNB*. The imperfect base-pairing between *lincRNA-p21* at sites in the coding and untranslated regions of its targets, triggers the association of these mRNAs with the translational repressors RCK and FMRP leading to attenuation of their translation [109]. Both CTNNB1 and JUNB are pro-survival proteins [110,111]. Their silencing by *lincRNA-p21* dictates its tumor-suppressive functions, which have been elucidated in other cancer types as well [112,113]. 

**Figure 2:**
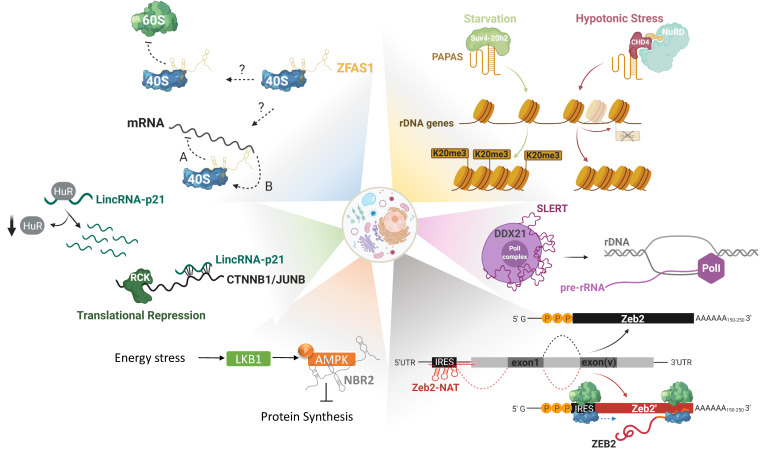
LncRNAs in translation regulation. In the nucleus, lncRNAs *PAPAS* and *SLERT* regulate ribosome biogenesis through regulation of rRNA transcription, while *Zeb2-NAT* by base-paring to its sense coding gene *Zeb2*, causes inclusion of an IRES thus leading to its translation. In the cytoplasm, lncRNAs *ZFAS1* binds the small ribosomal subunit and may have a role in mRNA translation. *NBR2* attenuates global translation by interacting with AMPK and promoting its kinase activity. *LincRNA-p21* regulates specific mRNA-targets by promoting their interaction with the translational repressors.

Apart from selective translational regulation of mRNA-targets there is accumulating evidence that lncRNAs can have broader effects on translation by regulating ribosome biogenesis in the nucleoli. There, lncRNAs can affect nucleolar structure, rRNA and/or ribosomal protein-transcription and maturation (Figure 2) [26]. Apart from the aforementioned lncRNA *PAPAS*, there are more lncRNAs implicated in ribosome biogenesis regulation. *SLERT* (SnoRNA-ended lncRNA enhances pre-ribosomal RNA transcription) is a box H/ACA small nucleolar RNA (snoRNA)-ended lncRNA, expressed in embryonic stem cells but also in several human cancer cell lines. These box H/ACA snoRNAs are present at both ends of the lncRNA and are required for its biogenesis and translocation to the nucleolus. *SLERT* physically interacts with and sponges the DEAD-box RNA helicase DDX21, a protein that coats polymerase I complexes blocking pre-rRNA transcription. This interaction allows Pol I to freely transcribe pre-rRNAs, making *SLERT* a positive regulator of ribosome biogenesis [114]. In keeping with this, *SLERT* inhibition reduces tumorigenic potential both *in vitro* and *in vivo*, thus acting as an oncogene.

After ribosome biogenesis in the nucleoli, ribosomal subunits have to be exported to the cytoplasm in order to actively engage in translation. Indications that lncRNAs could bind and modulate the activity of ribosomes themselves are starting to emerge. One such lncRNA could be *ZFAS1,* initially discovered in murine tissues undergoing mammary gland development and later on shown to be highly expressed in breast cancer, among others. Utilizing polysome profiling, Hansji and colleagues demonstrated that *ZFAS1* associates with the 40S small ribosomal subunit [115]. Even though mechanistic insights are still missing, it is intriguing to speculate that lncRNAs could serve as general modulators of protein synthesis initiation, either through modulation of ribosome assembly or via regulation of translation initiation factors (eIFs), elongation, and termination. 

All these layers of regulation do not only take place at the steady-state but are actually exacerbated under stress conditions where cells are forced to attenuate global translation in the effort to save energy [116]. Under nutrient-rich conditions, global translation is dictated by the recognition of the 7-methylguanosine (m7Gppp) in the 5’-cap structure of mRNAs by eukaryotic initiation factor 4E (eIF4E) [117]. Translational reprogramming occurs upon environmental cues when the cells cannot meet the energy demand and in effort to survive, they shut-down global translation while still maintaining protein synthesis of essential factors for survival, through alternative mechanisms. The most common ways to activate these alternative routes that are cap-independent, are by recognition of upstream open reading frames (uORFs) and internal ribosome entry sites (IRES) [118]. Although more light still needs to be shed on the contribution of lncRNAs on the regulation of translation rewiring, there are already some indications that they could be implicated in this dynamic process. One lncRNA that could be linked to it is *Zeb2-NAT* (Zeb2-natural antisense transcript). The protein *Zeb2* is a transcriptional repressor of E-cadherin and regulator of epithelial-to-mesenchymal transition (EMT), a process vital during physiological development and cancer progression [119]. *Zeb2* contains an IRES sequence in an intron upstream of its first exon and alternative splicing of this intron is actually regulated by *Zeb2-NAT*. Upon induction of EMT the lncRNA *Zeb2-NAT* physically binds and masks the splicing site of the IRES, leading to intron retention. Under these conditions, *Zeb2* is translated through the IRES, thus leading to downregulation of its target E-cadherin [120]. *Zeb2*-NAT’s ability to control a fundamental process as EMT has been shown to favor the maintenance and metastasis of some human cancers [121,122]. 

A last, indirect, method to regulate translation is the modulation of signaling pathways controlling it. The lncRNA *NBR2* (neighbor of *BRCA1* gene 2) is induced upon energy stress by the LKB1-AMPK pathway, with AMPK being a major sensor of the cellular energy levels. Upon such a stress *NBR2* physically interacts with AMPK promoting its kinase activity, signaling towards attenuation protein synthesis and activation of pathways that will restore the energy levels [123]. Depletion of *NBR2* leads to unchecked cell cycling, perturbed apoptotic/autophagic responses, and increased tumor development *in vivo* [124].

## 3. LncRNAs in Tumorigenesis

**Figure 3:**
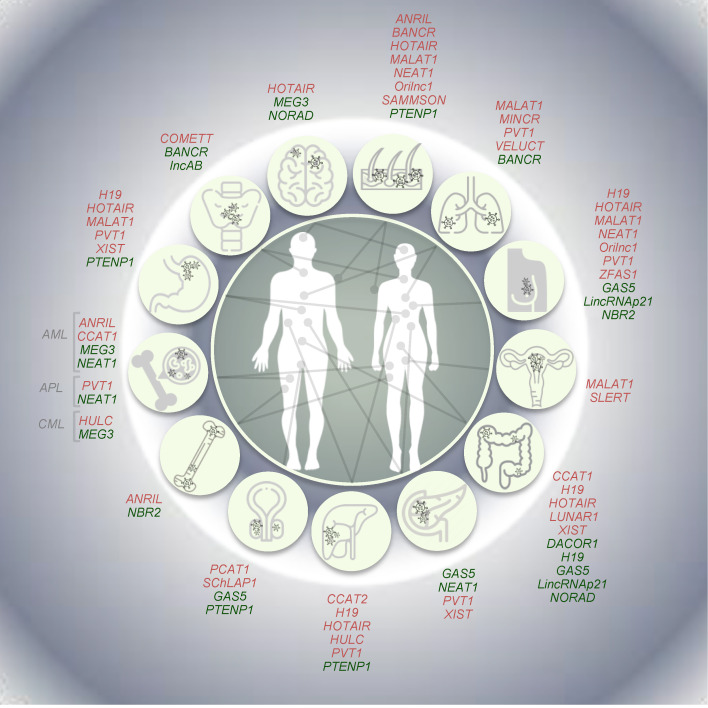
LncRNAs involved in different cancer types. Main examples of lncRNAs with oncogenic (red) or tumor-suppressive (green) properties reported in different tumors (i.e., clockwise from top left: neuroblastoma, melanoma, lung c., breast c., ovarian c., colon c., pancreatic c., hepatocellular c., prostate c., osteosarcoma, leukemia, gastric c., papillary thyroid c.). AML = acute myeloid leukemia; APL = acute promyelocytic leukemia; CML = chronic myeloid leukemia.

Systematic analyses based on TCGA data ([125] among the others) and the Cancer LncRNA Census (CLC; [126]) have largely contributed to reveal tumor-specific expression signatures of lncRNAs in cancer. Afterwards, different independent studies based on strong functional, genetic, and evolutionary evidence have reported, for a minority of them, a causative role in cancer. Hereafter, we provide—with obvious limitations due to space constraints—a description of the most relevant examples of well-established lncRNAs having oncogenic, tumor suppressive or dual properties, also reporting examples of lncRNAs whose expression is driven by well-known oncogenes (Figure 3).

### 3.1. Oncogenic LncRNAs

Among several oncogenic lncRNAs, here we focused on *MALAT1 and HOTAIR,* whose oncogenic potential is mainly related to tissue metastasis, as well as on *SAMMSON* and *VELUCT,* especially associated with sustained proliferation and abnormal cell metabolism, respectively. Furthermore, some examples of oncogenic lncRNAs induced downstream of the activation, e.g., by point mutations or rearrangements, of known oncogenes (i.e., *BRAF, RET*, and *MYC*) and/or regulating the expression of known oncogenes, such as *COMETT*, are also described below. 

The lncRNA metastasis-associated lung adenocarcinoma transcript 1 (*MALAT1*) was first identified as aberrantly expressed in metastatic lung cancer [127]. *MALAT1*, which is one of the most abundant lncRNAs in the cell, localizes to nuclear speckles, where it interacts with serine-rich (SR) proteins, regulating their abundance in the nucleoplasm and their phosphorylation levels, thus affecting alternative splicing [128]. A relevant role in the epigenetic regulation of transcriptional programs has been reported. Indeed, the relocation of growth control genes in the three-dimensional space of the nucleus—from repressive polycomb-enriched bodies (PcGs) to transcription-permissive interchromatin granules (ICGs)—is driven by the binding of *MALAT1* and polycomb 2 protein [129]. Mouse xenograft-based studies revealed an oncogenic activity of this lncRNA, as the metastatic properties of injected lung cancer cells were dramatically reduced by its deletion [130]. Despite being initially identified for its oncogenic role in lung, *MALAT1* expression was found deregulated in several tumors, including neoplasms of the digestive system [131,132,133] and ovarian cancer [134]. In keeping with this, *MALAT1* knockdown has been proposed as a promising strategy to block the metastatic capacity of breast cancer [135]. Stable or transient KD in breast tumor cells resulted in a significant reduction of tumor growth as well as in dramatic impairment of metastasis formation, associated in vivo with increased differentiation state and consequent formation of cystic tumors [135]. Furthermore, somatic mutations in *MALAT1* have been so far identified in women with breast cancer (luminal-type) [136] and its depletion in luminal cells from ER+ tumors has been reported to dramatically restrain tumor cells’ proliferation [137]. This evidence supports its KD as a valuable resource, especially in breast cancer treatment.

The human *HOX* transcript antisense intergenic RNA, known as *HOTAIR*, is embedded in the *HOXC* locus and was firstly described as interacting with EZH2 and SUZ12, members of the polycomb repressive complex 2 (PRC2) exerting its function *in trans* [138]. Interestingly, the cognate mouse *Hotair* gene (*mHotair*) is poorly conserved in the mouse genome and has a different exon number with highly variable sequence similarity [139]. In addition, it lacks the binding sites for EZH2, responsible for its methyltransferase activity and typical of the human *HOTAIR* lncRNA. Indeed, *HOTAIR* recruits the lysine specific demethylase (LSD1), part of CoREST/REST complex responsible for gene repression by chromatin remodeling. Adopting a secondary structure, human *HOTAIR* acts as a scaffold between these complexes inducing the formation of a transcription-repressive chromatin state around *HOXD* locus, which causes its silencing [68,72,140,141]. Conversely, no detectable regulatory effect of *mHotair* on *Hoxd* cluster genes was observed in mice, suggesting the presence of redundant mechanisms, cell type-specific functions or a rapid evolution of this lncRNA [139]. An oncogenic role for *HOTAIR* has been reported in several tumors and, consequently, this lncRNA is considered a potential therapeutic target in different cancer types. Indeed, its activity has been associated with increased invasiveness and metastatic capacity in primary breast and colon tumors, where high levels of *HOTAIR* positively correlate with metastasis and poor outcome [130,142]. Moreover, it is considered an independent prognostic marker in hepatocarcinoma for recurrence and impaired survival [14,129].

The survival associated mitochondrial melanoma specific oncogenic non-coding RNA (*SAMMSON*) maps to human chr3p13, a region amplified in 10–15% of human melanomas that also hosts the transcription factor *MITF*, the master regulator of melanocyte and melanoma biology [143]. Melanoma-specific expression of *SAMMSON* is thought to increase cancer cell fitness by concertedly enhancing mitochondrial and cytosolic translation via the sequestration of the nuclear RNA-binding protein CARF in the cytoplasm, where it makes aberrant contact with the mitochondrial protein p32 [144]. Of note, the primate-specific lncRNA *SAMMSON* is detectable in more than 90% of melanoma patients, but not in normal adult tissues, and its silencing with antisense oligonucleotides (ASO) results in the potent induction of cell death and increased sensitivity to MAPK inhibition both in vitro and in patient-derived xenograft models (PDX). Therefore, targeting *SAMMSON* represents a new promising cancer-specific therapeutic option in melanoma [143]. 

Then, viability enhancing lung cancer transcript (*VELUCT*) is a relevant example of extremely low-abundance lncRNAs. Despite this, silencing *VELUCT* lncRNA in lung cancer cell lines causes a dramatic drop of viability, indicating a potential oncogenic role for this low-copy lncRNA [145]. Novel opportunities for therapeutic strategies are also arising from the identification of lncRNAs whose expression is induced by oncogenes and are able to modulate their activity. For instance, *PVT1, CCAT1, CCAT2, PCAT1*, and *MINCR* lncRNAs have been identified among the most promising lncRNAs regulating Myc activity in tumor cells [146,147,148,149,150]. These lncRNAs map in 8q24– in a so-called “gene desert” (due to the lack of protein-coding genes), close to the proto-oncogene *MYC.* The identification of enhancers physically interacting with *MYC* and the amplification of this genomic region in different cancer types highlighted the potential role of lncRNAs mapping to 8q24 [151,152]. Among them, the lncRNA plasmacytoma variant translocation 1 (*PVT1*), discovered as an activator of Myc [153,154], is encoded by the *PVT1* gene in humans. This lncRNA is located 51 kb downstream of the *MYC* locus (RefSeq on hg38 genome release) [146,155] and is co-amplified in a variety of human and animal tumors, which results in Myc stabilization. Furthermore, *PVT1* is often involved in DNA rearrangements (e.g., translocations in Burkitt lymphoma, fusion genes with *NBEA* and *WWOX* in multiple myeloma, *APIP, ATE1,* and *PPAPDC1A* in the human gastric cell line), which could activate Myc by disrupting the gene structure or protein production [146,155,156,157,158,159,160]. Moreover, *PVT1* has been demonstrated to be a MYC target and to be part of the same ribonucleo-protein complex [160]. However, although the role of *PVT1* in tumorigenesis has been defined as dependent on increases in the MYC copy-number, an independent contribution to tumorigenesis has also been proposed [161]. Indeed, *PVT1* inhibition can induce cell apoptosis, even in *PVT1*-overexpressing cells, whereas MYC silencing does not produce the same effect [162,163], suggesting different mechanisms of *PVT1* and *MYC* cooperation in different cancers. Of note, the over-expression of *PVT1* was reported in a wide range of cancers, including breast, gastric, non-small cell lung cancers (NSCLC), pancreatic and hepatocellular carcinomas, and acute promyelocytic leukemia (APL), and serves as a predictor of tumor progression and prognosis [161,163,164,165,166,167].

Likewise, prostate cancer associated transcript 1 (*PCAT1*) is another lncRNA mapping approximately 710 kb upstream of the *MYC* gene (RefSeq on hg38 genome release) and responsible for its post-transcriptional regulation. Identified in prostate cancer as a posttranscriptional repressor of *BRCA2* [149], *PCAT1* also acts as competitive endogenous RNA (ceRNA) by sponging miR34-1 and abrogating its binding to *MYC* 3’UTR, thus increasing Myc protein levels [168].

Furthermore, the colon cancer-associated transcripts 1 and 2 (*CCAT1* and *CCAT2*, respectively), mapping upstream of the *MYC* locus at ≈515 kb and 333 kb, respectively (RefSeq on hg38 genome release) have been also reported to regulate Myc [147,169]. Interestingly, their expression correlates with a cancer-associated single nucleotide polymorphism (SNP), the rs6983267, which is located in a super-enhancer around the transcription start site of the *MYC* gene at 8q24. The silencing of *CCAT1* in colon cancer cells reduces proliferation through CDKN1A/p21-mediated cell-cycle arrest in G1 phase and the injection of *CCAT1* KD tumor cells delays tumorigenesis in xenograft models [148]. Moreover, *CCAT1* is aberrantly upregulated in patients with acute myeloid leukemia (AML) and promotes cell proliferation by regulating miRNA-mediated pathways [170]. Conversely, over-expression of *CCAT2* lncRNA, a target of Wnt-signalling, increases the expression of *MYC* and some of the members of the miR-17-92 cluster through TCF7L2 transcriptional regulation. This event promotes invasive tumor growth in xenograft models, conferring tumor cells with higher metastatic capacity in the liver [147].

Finally, the Myc-induced lncRNA *MINCR* was identified in Burkitt lymphoma cells as contributing to Myc-mediated regulation of cell cycle genes and having a relevant role in cell cycle progression [150]. Indeed, *MINCR* silencing causes the downregulation of *AURKA* and *AURKB* genes, encoding the Aurora kinase A and B, respectively, and of the chromatin licensing and DNA replication factor 1 (*CDT1*), determining a dramatic reduction in cell proliferation. Furthermore, *MINCR* overexpression was assessed in NSCLC patients and cell lines where its silencing induces cell cycle arrest and apoptosis by reducing Myc expression and its downstream effectors (e.g., cyclin A, cyclin D, CDK2, Bcl-2) [171]. 

However, lncRNAs induced by other oncogenes have also been reported, as the oncogenic RAS-induced lncRNA 1 (*Orilnc1*)—identified in breast and melanoma cells as being induced by mutated BRAF [172]—and the cytosolic oncogenic antisense to *MET* transcript (*COMETT*), over-expressed in *BRAF*-mutated and *RET*-rearranged papillary thyroid carcinomas [173]. Zhang and colleagues reported that *Orilnc* silencing by shRNAs causes a down-regulation of Cyclin E1 inducing G1/S arrest and blocking tumor cell proliferation and growth, both in vitro and in vivo [172]. Similarly, the repression of *COMETT* markedly reduces the viability and proliferation of tumor cells harboring *BRAF* mutation or *RET* oncogene rearrangement, as well as the motility and invasiveness of tumor cells in vitro. Of note, *COMETT* depletion significantly impairs the expression levels of different MAPK pathway effectors, including—but not limited to—the *MET* oncogene, also increasing tumor cells’ sensitivity to vemurafenib, a common inhibitor of mutated B-raf [173].

### 3.2. Tumor Suppressive lncRNAs

An increasing number of studies are reporting lncRNAs acting as tumor suppressors, whereby their inactivation/deletion in tumors has a role in the onset and/or progression toward metastatic advanced cancer stages. Here, we discuss five lncRNAs whose tumor-suppressing activities have been reported in different tumor types. 

Among highly expressed lncRNAs, the growth arrest-specific transcript 5 (*GAS5*) has been reported to be ubiquitously expressed according to serial analysis of gene expression (SAGE) in cancer as well as normal tissue [174]. Well-known for its role in embryogenesis [175], it also plays a key role in other relevant processes, such as p53 signaling [176], growth arrest [177], and apoptosis [178]. One of the reported roles for *GAS5* lncRNA is to act as a decoy for the glucocorticoid receptor [179] even though it can also bind to the receptors of androgen and progesterone, having a role in tumors with hormone-dependent induction [175]. *GAS5* expression levels are inversely correlated with tumor size, staging, and also metastasis in different tumor types, including breast, bladder, colon, pancreas, and prostate cancer [180,181]. For instance, its overexpression in xenograft mice models of breast cancer significantly reduces tumor growth in vivo through cell-cycle arrest and induction of apoptosis [178].

Discovered as the human ortholog of gene trap locus 2 (*Gtl2*) in mice, the lncRNA maternally expressed gene 3 (*MEG3*) is a member of the imprinted genes mapping at 14q32.3 [182,183]. This lncRNA activates p53 and its target genes, showing a tumor suppressor activity related to its ability to inhibit tumor cell proliferation, as shown in various cancer cell lines following in vitro re-expression [184,185,186,187,188]. However, in AML and chronic myeloid leukemia (CML), it has been reported that *MEG3* inhibits cell growth also in a p53-indipendent manner [189,190]. According to its tumor-suppressive activity its expression is lost in primary tumors, i.e., neuroblastomas, gliomas, and cell lines, e.g., those derived from brain, bladder, bone marrow, breast, colon, liver and prostate, owing to different mechanisms [184,185,186,187,188,189,190,191,192]. Deletion is the most frequent mechanism for *MEG3* silencing in tumor cells. However, its negative epigenetic regulation is very frequent in cancers. *MEG3* lncRNA maps in a genomic locus enriched in CpGs, highly sensitive to methylation. In tumors, the hypermethylation of *MEG3* promoter and hypomethylation of the surrounding intergenic region have a pronounced effect on its expression levels [185]. The loss of *MEG3* expression has also been associated with tumor grade in meningiomas [186]. Independent studies assessed *MEG3* ability to inhibit tumor cell proliferation and induce cell apoptosis in different cell lines [184] [187,188]. Furthermore, *MEG3* inactivation leads to an increased expression of genes promoting angiogenesis and microvessel formation in the brain [192].

The lncRNA AB074169 (*lncAB*) is a single-exon lncRNA first identified in a comparative microarray analysis as down-regulated, due to promoter hypermethylation, in papillary thyroid carcinoma samples when compared to adjacent non-tumor counterparts [193]. Accordingly, its knockdown promoted tumor cell proliferation in vitro. Conversely, its forced over-expression resulted in cell-cycle arrest and tumor growth inhibition in vitro and in vivo. Mechanistically, *lncAB* binds to, and reduces the expression of, *KHSRP*, increasing p21 levels and simultaneously decreasing *CDK2* expression, in turn repressing cell proliferation [193].

The lncRNA Non-coding RNA Activated by DNA damage (*NORAD*), previously annotated as LINC00657, is conserved and ubiquitously expressed, although RNA-Seq data from normal post-mortem brains collected in GTEx database (https://www.gtexportal.org/home/gene/NORAD) indicate NORAD as highly expressed in frontal cortex. NORAD expression is triggered in a p53-mediated manner by DNA damage [194]. It has been also reported that *NORAD* acts as decoy in colon cancer for the members 1 and 2 of the RNA-binding proteins belonging to the PUMILIO family [195]. Proteins of this family stimulate deadenylation and decapping of messenger RNAs leading to post-transcriptional repression. In the absence of *NORAD,* the chromosomal instability is induced by the repression of their targets which, among other things, include many genes encoding proteins crucial for DNA replication and repair processes, affecting in turn the cell cycle and mitosis [194].

Tumor protein P53 pathway corepressor 1 (*TP53COR1*), commonly known as *lincRNA-p21*, was identified as highly transcribed upon DNA damage in a p53-mediated manner [196]. *LincRNA-p21* is an antisense transcript of *CDKN1A*, the tumor-suppressor gene known as p21. It is a transcriptional repressor which is induced in p53-dependent transcriptional responses [176,196]. *CDKN1A* expression is affected by *lincRNA-p21* silencing which causes the activation of the protein-coding gene *in cis*. As reported by Dimitrova and colleagues it causes the disruption of G1/S cell-cycle checkpoint [176]. In addition, *lincRNA-p21* expression correlates with tumor staging and invasive phenotype in colon cancer [197]; it interacts with HuR in breast cancer cells causing the transcriptional repression of *CTNNB1* and *JUNB* genes [109]. Finally, *lincRNA-p21* is one of the few lncRNAs regulating tumor metabolic rewiring. Indeed, it has been reported that it is transcriptionally induced in hypoxic conditions and its increase is associated with the induction of hypoxia-induced glycolysis in tumor cells [198].

### 3.3. LncRNAs with Dual Activity

A few cancer-associated lncRNAs have been reported to play divergent roles in different cancer types. These contrasting functions could be explained, at least in part, by the wide genetic and phenotypic heterogeneity of tumors. Thus, tumor- or microenvironment-specific factors could determine oncogenic or tumor suppressor activities of a given lncRNA. However, the use of distinct experimental systems/approaches (e.g., in vivo models vs. in vitro studies) could also contribute to the generation of confounding results. Some clear and well-known examples of lncRNAs with apparently divergent roles in tumors are provided here.

The lncRNA *H19* has been discovered for its high expression along mouse embryogenesis. It is a paternally imprinted gene mapping at chr11p15.5. As such, it is repressed at birth in most tissues. H19 expression is induced in cancer cells due to a loss of imprinting at its locus [199,200], especially in breast, colon, and hepatic carcinomas [201,202]. Its transcriptional regulation is exerted by the oncogenes p53, Myc, and hypoxia-induced factor 1a (HIF-1a) which are frequently deleted (p53) or over-expressed/amplified (Myc and HIF-1a) [203,204,205]. RNA-Seq data collected in the Cancer Genome Atlas portal report *H19* lncRNA to be over-expressed in colon and gastric cancers [206]. However, a tumor-suppressive role has been reported in colon cancer mouse models as well as in human rhabdoid tumors [207,208,209]. The genetic heterogeneity of tumors and the intrinsic inter-species differences may account for discrepant results. *H19* exerts its activity on tumor progression by different mechanisms. It can act as ceRNA for a large subset of microRNAs and it can simultaneously be a precursor of miRNAs. Its activity as a miRNA "sponge", in line with its main localization in the cytosol, has been reported for let-7 and miR-200 family members [210]. However, *H19* is also a miR-675 precursor [208]. In colon cancers, the over-expression of *H19* is paralleled by miR-675 increase. This miRNA directly binds and inhibits the tumor-suppressor retinoblastoma (RB) protein, promoting cell proliferation [211].

The MENepsilon/beta on chromosome 11 is a direct target of p53 [17] and gives rise to two transcriptional variants of the nuclear enriched abundant transcript 1 (*NEAT1*) non-coding RNA, a short polyadenylated (3.7 kb) and a long non-polyadenylated (27 kb). The last one is essential for the formation of paraspeckles, i.e., nuclear membraneless bodies [212]. The switch between the two isoforms was recently ascribed to the integrator complex that favors the production of the short form over the long [27]. Furthermore, *NEAT1* long form—and, thus, the paraspeckles—promote skin tumorigenesis by increasing survival of tumor cells expressing mutated *KRAS* in genetically engineered mouse models [17]. In addition, its knockdown synergizes with several genotoxic chemotherapeutics, suggesting *NEAT1* levels as good a predictor of responses to chemotherapy [17]. In keeping with its oncogenic role, mutations in CpG islands within *NEAT1* promoter have been reported in breast cancer [213], where increased levels of *NEAT1*—and paraspeckles—correlate with staging in HER^+^ patients [214]. Nevertheless, the Attardi’s lab reported instead a tumor suppressor role for *NEAT1* in p53-/- mutant *KRAS* mouse model of pancreatic ductal adenocarcinoma [215]. Accordingly, reduced *NEAT1* expression was reported in AML and APL [216,217]. However, whether the tumor suppressive function of *NEAT1* stems from the long or the short transcript needs to be further investigated. Due to the nature of paraspeckles, which are able to sequester other biomolecules in the nucleus, and to the difference in the germ layer originating from the affected tissue (ectoderm for skin and endoderm for pancreas), a context-specific function for this lncRNA it is very likely.

The *BRAF*-activated lncRNA (*BANCR*) has been reported either for its oncogenic or tumor suppressive activity. First identified as an oncogene in melanoma for its ability to activate MAPK pathway and induce tumor cell migration and proliferation [218,219], *BANCR* has also been reported as tumor suppressor in NSCLC for its role in epithelial-mesenchymal transition (EMT) [220]. In line with this finding, down-regulation of *BANCR* is associated with increased metastatic potential of tumor cells and poor prognosis [220]. Likewise, its down-regulation has been positively associated with tumor progression in papillary thyroid and clear cell renal cell carcinomas [221], indicating—at least in these cancer types—a tumor suppressive role.

## 4. Strategies to Target lncRNAs

Technical advances in molecular therapies provide new opportunities for exploring the clinical relevance of lncRNAs. The identification of lncRNAs involved in tumorigenesis provided useful insights in cancer biology, also paving the way to the adoption of new therapeutic strategies based on their targeting—e.g., CRISPR/dCas9-based approaches [222]—and to their evaluation as novel diagnostic/prognostic markers. Especially for lncRNAs with direct or indirect oncogenic activity, different targeting strategies have been adopted by modulation at the (i) genome-level (i.e., affecting their transcription levels), (ii) post-transcriptional RNA-level (i.e., regulating lncRNAs stability and/or processing) and (iii) interaction-level (i.e., modulating their binding capacity to obtain steric inhibition of specific lncRNA-protein interactions).

### 4.1. Genomic Modulation of LncRNAs by CRISPR-Based Systems

Targeting strategies aimed to modulate lncRNAs activity by genomic regulation benefitted from recent advances in genome editing approaches based on CRISPR-based systems. Particularly, among the systems of the so-called class 2–which use single effectors—the type II CRISPR-system, in its simplest form, consists of a nuclease (Cas9) and a single guide RNA (sgRNA), which leads Cas9 to target precise sites of the DNA [223]. Its main applications consist of the knockout of target genes by the generation of double-stranded breaks (DSB) in the open reading frame and the following induction of the non-homologous end joining (NHEJ) repair system (systems termed CRISPRn mutagenesis), as well as gene corrections or overexpression by inducing homology-directed repair (HDR; CRISPRn HR) or genomic deletions following multiple DSB (CRISPRn excision) [223,224,225]. However, the intricate genomic architecture of lncRNAs, the incomplete knowledge of their promoters and/or interaction motifs, as well as the complex mechanisms of their multiple activities (*in cis* and *in trans*) limit the use of standard CRISPR-based approaches. Of note, Goyal and colleagues [224] characterized about 62% of the total lncRNAs as “non-CRISPRable”- due to the presence of internal (35%) or bidirectional (20%) promoters–and only 38% of lncRNAs as specifically targetable.. Indeed, classical systems based on induction of frameshift mutations (e.g., the CRISPRn method) could be applied for modulating lncRNAs activity only if the sequence of the recognized motif is completely characterized and additional mechanisms of action are excluded. Additionally, targeting lncRNA promoters (e.g., by CRISPRn or HDR-based CRISPR approaches) needs a complete knowledge of the nucleotide sequences and could however affect the expression of other genes due to potential overlap and interactions between the promoters, or due to their proximity [226,227,228]. Furthermore, promoter targeting may also be insufficient if other, unknown, internal promoters contribute to the regulation of their expression. Similarly, the total or partial excision (e.g., by CRISPRn system) could be unfeasible if the lncRNA sequence overlaps with other functional regions or could lead to the generation of DNA artifacts as newly combined elements [225,227,228].

Interestingly, innovative CRISPR approaches have provided powerful tools to overcome classical editing allowing to specifically and reliably modulate lncRNAs expression (reviewed in [225,229]). Among the most prominent methods are the double excision CRISPR knockout (DECKO) system and the combination of CRISPR interference (CRISPRi) and "dead"-Cas9 (dCas9) [225,228,230,231,232]. More in detail, dCas9 lacks the nuclease activity but still preserves its RNA-dependent DNA-binding activity. Its fusion with specific effector domains has allowed the development of additional systems for gene expression modulation [224,230]. For instance, dCas9 fusion to the KRAB (Krüppel-associated box) domain of ZNF10 allows a more robust transcription inhibition (CRISPRi), whereas targeted gene expression *in cis* is induced by its combination with activation domains, such as VP64, p65, or Rta (CRISPR activation, CRISPRa) [224,230]. Of note, the CRISPRi system was employed for targeting promoters of more than 16000 lncRNAs and revealed that about 500 of them have causal roles in cancer cell growth [232,233]. Furthermore, the silencing of different lncRNAs (e.g., *MALAT1*) was achieved by the CRISPR/Cas9 system and the insertion of an inhibitory signal [234,235]. Even the combination of CRISPR-dCas9 to epigenetic effector proteins (such as DNA methyltransferase or histone modifiers) [236] has been successfully used for epigenetic silencing of lncRNAs. For example, the epigenetic silencing of *MALAT1* was reached by excising active histone marks associated to its TSS [237]. Then, a good applicability for targeting lncRNAs has also been showed for CRISPR-based targeted insertion utilizing the NHEJ pathway—given the absence of a homologous donor sequence [238,239]—as well as for the DECKO method, which applies a two-step cloning strategy to generate lentiviral vectors expressing simultaneously two guide RNAs (gRNAs) [228].

The manipulation of lncRNAs expression has been reported also by the CRISPR-mediated tagging and regulation of lncRNAs (CTRL) method, consisting of a modified gene trap vector and a plasmid with Cas9 and 2 sgRNA sequences [240]. This approach—successfully applied for upregulating *HOTAIR*, *DICER1-AS1* and *PTENP1*, among others—could represent a valuable tool for future screening of several lncRNAs [225,240].

Furthermore, novel Cas enzymes are starting to be utilized, such as the CRISPR/Cas13 system (class 2, type VI). It has showing remarkable potential for the development of basic and clinical approaches in cancer, as well as fortargeting lncRNAs [241,242,243]. Particularly, type VI enzymes possess RNA recognition and cleavage activities and, following the formation of guide-target RNA duplexes, it binds and cleaves targeted complementary sequences producing efficient gene knockdown without genome manipulation [241,244]. Thus, instead of the DNase activity of Cas9, Cas13 holds RNase activity and the distinct subtypes (i.e., Cas13a, Cas13b, Cas13c, and Cas13d), although different in sequence and size, share as common features the presence of higher eukaryotes and prokaryotes nucleotide-binding (HEPN) domains, which account for the RNA-targeted nucleolytic activity [245]. CRISPR/Cas13 systems have been successfully employed for multiple applications (e.g., RNA knockdown, imaging, tracking, and editing). Notably, its high sensitivity and specificity has been also assessed for lncRNA knockdown in a high-throughput phenotypic assay based on survival challenge in anticancer drugs response and the targeting of a subclass of nuclear lncRNAs—i.e., very long intergenic non-coding (vlinc) RNAs—by Cas13a from *Leptotrichia wadei* (LwCas13a) [243]. Furthermore, the catalytically “dead” Cas13 system (dCas13) has been recently used for labeling *NEAT1* lncRNA and to explore the dynamics of associated paraspeckles without inducing genome alterations [242].

However, despite the remarkable advance in the development of innovative CRISP-based systems, the accurate analysis of lncRNA loci (for designing specific RNA guides), the evaluation of expression of neighboring genes and the validation of results by other approaches (e.g., RNAi, ASOs) should be implemented when targeting lncRNAs by CRISPR editing approaches. Future studies will be instrumental for assessing the translational potential of CRISPR-based targeting strategies of lncRNAs in cancer therapies.

### 4.2. Post-Transcriptional Targeting of LncRNAs

Oligonucleotide-based approaches which depend on RNA-RNA or RNA-DNA duplex formation have been developed as a strategy for the successful post-transcriptional targeting of long non-coding RNAs [246,247]. Double-stranded small-interfering RNAs, i.e., dssiRNAs, represent the most commonly used approach for lncRNA silencing. It is based on Dicer and the RNA-induced silencing complex (RISC) degradation pathway and involves the Argonaute 2 (Ago2) protein [248]. This approach has proven extremely successful in targeting lncRNAs for degradation in many cancer cell lines, although in vivo experiments using siRNAs have been even more challenging. However, their limited bioavailability and susceptibility to nucleases has largely restricted their use for in vivo purposes. However, to prevent siRNAs from enzymatic degradation, some chemical modifications (e.g., 2′-O methyl sugar residues and phosphorothioate bonds at the 3′-end) have been adopted to improve their pharmacokinetic properties [249]. This is the case of patisiran (Alnylam Pharmaceuticals) - a double-stranded siRNA comprised of a 3’-end DNA, RNA, and 2’-O-MOE RNA moieties, used for inhibiting the hepatic synthesis of transthyretin and approved by FDA for the polyneuropathy of hereditary transthyretin amyloidosis [250].

An alternative approach developed more than three decades ago is represented by the antisense oligonucleotides (ASOs) [251]. These were designed for the first time as short (13-mer) single strand DNA (ssDNA) oligos in order to target the Rous sarcoma virus RNA. Since their first use, ASOs have been widely adopted in many studies to successfully modulate lncRNA levels. ASOs show some differences compared to siRNAs. Indeed, the former are single-stranded DNA oligonucleotides (13–15 bp) which bind, via standard Watson-Crick base-pairing, target nucleotides in the RNA of interest. The formation of DNA-RNA duplex causes the degradation of the target RNA by RNAse H, even though specific ASO can act by inhibiting or modifying the expression of their target via steric hindrance and/or they can modulate splicing. However, the most useful feature of ASOs is the capacity to specifically target nuclear RNAs and, due to RNase H enrichment in the nucleus, cause its degradation. For this reason, ASOs are currently employed to achieve significant knockdown of lncRNAs [252]. As described for siRNAs, new generations of ASOs, consisting of longer (15–20 nt) oligos with phosphorothioate modifications, have been developed. These improvements have significantly increased ASO stability, making these oligonucleotides even more resistant to endonuclease-mediated degradation [253,254]. However, in the last two decades new chemical modifications, including 2′-O-methoxyethylation, have been used to maximize the RNAse H-mediated degradation of target RNAs and the duplex stability as well as the resistance of free ASOs to endonuclease-mediated degradation. These modifications have also largely reduced the so-called “off-target” effects, conferring to ASOs with new drug-like features [255]. This new class of chimeric ASOs—commercialy known as Gapmers—consists of RNA-DNA hybrids carrying a 2′-O-methoxyethyl-modified sugar backbone. Other chemical modifications, such as the S-constrained ethyl (cEt) and the locked nucleic acids (LNAs), have finally improved ASO potency revealing new pharmacokinetic properties, which make these oligo-based approaches the most useful tools to target lncRNA both in vitro and in vivo.

### 4.3. Steric Inhibition of LncRNA-Protein Interactions

Different experimental evidence has demonstrated that lncRNAs can act as scaffolds for single or for multi-protein complexes due to their peculiar folding. As these ncRNAs mediate the interaction of regulatory DNA regions, RNAs, and binding proteins, interfering with their binding can provide new therapeutic solutions. Targeting lncRNA unique structural regions by ASOs represents one of the most reliable approaches. In particular, the adoption of modified ASOs, unable to trigger RNAse H-dependent degradation of RNAs (e.g., morpholinos), as well as of small molecules able to block contact interface/s between lncRNAs and proteins, can result in loss-of-function [256]. Indeed, extending the aforementioned chemical modifications (e.g., cEt and LNAs) to the entire sequence of the ASO would impede RNAse H-dependent degradation of the lncRNA, causing only the alteration of the splicing, as described for splice-switching oligos (SSOs). Indeed, the oligos act by blocking the binding of splicing regulatory elements, such as enhancers or silencers (by steric hindrance), and in turn modulate pre-mRNA splicing [257]. To date, new therapies based on splice-switching oligos have been approved by the FDA for the spinal [258] and Duchenne muscular atrophy [259]. 

Similarly to SSOs, morpholinos are non-ionic DNA analogs of about 25 oligonucleotides initially developed to block mRNA translation or promote splicing switch, very similarly to LNAs. These molecules, first used to study zebrafish development [260,261], received their first approval in the clinics as *DMD* splicing modulators in patients affected by Duchenne muscular dystrophy [262,263,264]. In morpholinos sugars are replaced with methylene morpholine rings whereas anionic phosphates of DNA/RNAs are substituted by non-ionic phosphorodiamidate bonds [261,265,266]. Due to these chemical modifications, the morpholinos abolish, or largely suppress, lncRNAprotein binding, also impeding lncRNA binding to gene regulatory regions of the DNA.

LncRNA ability to form stable secondary and tertiary structures with DNA, RNA and proteins has provided new opportunities to target them [267]. New assays, such as selective 2′-hydroxyl acylation by primer extension (SHAPE; [268]) and the psoralen analysis of RNA interactions and structures (PARIS; [269]), have been developed to specifically map lncRNA secondary/tertiary structures. Bacterial/viral riboswitches, having structural elements similar to cancer-associated lncRNAs, have been successfully targeted with small-molecule inhibitors [270]. Hence, it is likely that a similar approach—coupling large scale experimental assays as SHAPE and PARIS to the screening of small-molecule inhibitors—may be used to target cancer-associated lncRNAs.

The use of oligo-based approaches to target lncRNAs in cancer has provided interesting results. Nonetheless, new challenges need to be faced and technical caveats need to be addressed. Indeed, despite the fact that chemical modifications have improved oligo stability and largely reduced, if not abolished, their endonuclease-mediated degradation, one the most limiting factors is represented by their capacity to activate the innate immune response. The intrinsic characteristic of innate immunity protecting cells from exogenous RNAs by Toll-like receptors (TLRs) has largely limited oligo bioavailability [271]. First-generation oligos, including short interfering RNAs, have “CpG” motifs which are recognized by TLR9 and activate a TLR-dependent pro-inflammatory response [272]. Despite ASOs having specific chemical modifications and nucleotide sequences (CpG-poor), which minimize the activiation of these intracellular responses, they can still trigger a TLR-independent mechanism, such as the retinoic acid-inducible gene I-like (RIG-I) RNA helicases (RLHs), which elicit a pro-inflammatory response in the cytosol [272,273]. Upon activation of these pro-inflammatory pathways ASOs are entrapped into endosomes and their bioavailability—and in turn their capacity to target lncRNAs—is dramatically impaired [274]. In this regard, new oligo coniugating strategies need to be adopted and well-tolerated sequences need to be identified [275,276].

In addition, new strategies to avoid off-target effects need to be explored, but most importantly rigorous studies about oligo safety in humans need to be conducted before applying these approaches for cancer therapies.

Nine clinical trials (active, recruiting, or completed; https://clinicaltrials.gov) are currently testing signatures of lncRNAs in breast, lung, thyroid, or stomach tumors or single lncRNAs (*XIST*, *CCAT1* and *HOTAIR*, in AML, colorectal, and thyroid carcinomas, respectively) as possible prognostic/diagnostic biomarkers. One of them is further validating prognostic and predictive mRNA-lncRNA signatures for triple-negative breast cancer which could be used to classify patients into high- or low-risk of recurrence. This trial is also evaluating these signatures as predictors of chemotherapy efficacy (e.g., doxorubicin, cyclophosphamide, gemcitabine, and cisplatin). However, to the best of our knowledge, no clinical trials targeting lncRNAs in cancer are currently underway.

## 5. Conclusions

A growing body of evidence has largely established that lncRNAs play a crucial role in tumor onset, progression, and drug responses. The rapid advancement of sequencing-based technologies, paralleled by consolidated functional in vitro approaches, as well as animal and human-derived tumor models, is offering an unprecedented opportunity in oncology, revolutionizing the way cancer is diagnosed and treated. Due to the high specificity in their expression—often restricted to tumor cells—many lncRNAs are currently under evaluation as biomarkers or direct therapeutic targets in clinical trials, whereas others have been proposed as modulators of tumor response to therapy. In this regard, despite RNA-based strategies specifically targeting lncRNAs in vitro and in vivo (e.g., in cancer mouse models) having provided encouraging results, lncRNA-based therapeutic approaches have not yet reached the clinical stage and different challenges are still open. Among them, the level of expression of lncRNAs is a key factor both for diagnostics and therapeutics. Identifying lncRNA candidates whose expression can be easily quantified and monitored is a relevant point to develop easy-to-use diagnostic tests. In addition, detecting lncRNAs through non-invasive approaches (e.g., in blood, urine, or saliva) would significantly boost the use of lncRNAs in daily clinical practice. It is reasonable to speculate that future decision trees in oncology will be revised by integrating patient-specific omics data, such as whole-genome/targeted resequencing (for somatic and germline mutations/rearrangements) and RNA-Seq (for profiling coding and ncRNAs). Hence, lncRNA detection for diagnostic and prognostic purposes or their therapeutic targeting will become a daily practice for clinicians, boosting the perspectives of personalized medicine, and especially of precision oncology.
